# Adapting Andersen’s expanded behavioral model of health services use to include older adults receiving long-term services and supports

**DOI:** 10.1186/s12877-019-1405-7

**Published:** 2020-02-14

**Authors:** Jasmine L. Travers, Karen B. Hirschman, Mary D. Naylor

**Affiliations:** 1 Yale University School of Medicine, 333 Cedar Street, SHM I-456, PO Box 208088, New Haven, CT 06510-8088 USA; 2 Yale University School of Nursing, 333 Cedar Street, SHM I-456, PO Box 208088, New Haven, CT 06510-8088 USA; 3 NewCourtland Center for Transitions and Health, University of Pennsylvania School of Nursing, 418 Curie Blvd, Philadelphia, PA 19104 USA

**Keywords:** Long-term care, Aging, Healthcare services

## Abstract

**Background:**

Andersen’s Expanded Behavioral Model of Health Services Use describes factors associated with the use of long-term services and supports (LTSS). This model, however, has only been tested on the *intent* to use such services among African-American and White older adults and not the *actual use*. Given the increasing diversity of older adults in the U.S., the ability to conceptualize factors associated with *actual use* of LTSS across racial/ethnic groups is critical.

**Methods:**

We applied Andersen’s Expanded model in the analysis of 2006–2010 qualitative data using multiple methods to understand both the relevancy of factors for older adults who currently use LTSS vs. those who intend to use LTSS (as described in Andersen’s original exploration). We additionally explored differences in these factors across racial/ethnic groups and included Hispanic older adults in our analyses.

**Results:**

Four additional constructs linked with actual LTSS use emerged: losses and changes, tangible support, capability to provide informal support, and accessibility of informal support. Racial differences were seen in level of participation in decisions to use nursing home services (Not involved: 45% African-Americans vs. 24% Whites). Reports of LTSS use to avoid burdening one’s family were greater among White older adults compared to African-American older adults.

**Conclusions:**

Findings around decision-making and burden along with other constructs enhance our understanding of determinants that influence *actual* LTSS use and require targeted interventions.

## Background

Older adults age 65 years and over currently account for 15% of the US population [1]. More than 90% of older adults live with at least one chronic disease and 85% of this group experienced a physical limitation in 2014 [2–4]. It is expected that restrictions older adults face as a result of progressive diseases and functional deficits will continue to escalate with the aging and increased longevity of the Baby Boomer population [2].

Consequently, the need for long-term services and supports (LTSS) among those experiencing functional limitations is expected to increase dramatically [3]. LTSS provide assistance with basic activities of daily living (ADL; e.g., eating, walking, toileting, bathing, and dressing) along with instrumental activities of daily living (IADL; e.g., food preparation, shopping, and management of finances). These services may be provided in either institutional settings such as nursing homes (NHs) and assisted living (AL) or in non-institutional settings such as older adults’ homes. Services received from paid caregivers are termed “formal” [5] whereas those received from unpaid caregivers are termed “non-formal” LTSS.

Historically, White older adults have accounted for the majority of individuals utilizing formal LTSS, despite minorities having greater functional limitations [6, 7]. Limited access to funding for these services and discrimination by institutions providing LTSS have been reported as barriers to accessing formal LTSS by racial and ethnic minority older adults [8]. Consequently, this underrepresentation of racial and ethnic minorities in institutionalized LTSS settings in past years has limited our understanding of this minority group’s utilization behaviors related to use of LTSS and hindered our ability to carry out national and state planning for LTSS delivery that considers the needs and preferences of all racial and ethnic groups [9].

### New contribution

In the last decade, the use of LTSS in institutionalized settings by racial and ethnic minority older adults (i.e., African-Americans and Hispanics) has nearly tripled [10]. This rapid demographic shift may be attributed to: a decline in informal support as a result of changes in family structure, increased access to public funding for the care of racial and ethnic minorities in institutionalized LTSS settings, and a rise in the healthcare needs of racial and ethnic minority older adults [2, 11, 12]. Nonetheless, increased utilization of care in institutionalized LTSS settings among racial and ethnic minority older adults warrants a greater understanding of this group’s LTSS needs and preferences and provides an opportunity to ensure future LTSS policies overall are racially and ethnically inclusive. Patterns of LTSS use by racial and ethnic groups have been examined by Bradley and colleagues and subsequently reported as an expansion of Andersen’s behavioral model of health service use [13]. Their contribution, however, focused only on factors influencing intent to use LTSS among White and African-American older adults. Additionally, their model does not describe how intent to use LTSS differs by LTSS type [14].

To expand on existing knowledge, we tested Andersen’s expanded behavioral model of health service use to see how it performed across diverse racial/ethnic groups in the context of actual use of LTSS differentiated by three LTSS types. We then discuss how the expanded Andersen model may be adapted to present constructs that reflect the actual use of services by LTSS type when considering a racially and ethnically diverse group of older adults.

## Methods

### Conceptual framework

#### Andersen’s expanded behavioral model of health service use

Andersen’s expanded behavioral model of health service use is an augmentation of Andersen and Newman’s 1995 behavioral model of health service use [14]. This original model aimed to: understand how and why people use healthcare services, assess inequality in access to health services, and aid in the creation of policies that will allow for equitable access to care [15]. To predict or explain one’s use of healthcare services, the original model particularly focused on an individual’s predisposition to use acute healthcare services, enabling factors that facilitate use, and one’s perceived or influenced need for care. With this model, one is able to assess measures of access (e.g., equitable, inequitable, effective, efficient) as well as understand the environment (external or healthcare system) impacting access and utilization of healthcare services. Outcomes describing health and consumer satisfaction are additional constructs important to the model.

The purpose of Andersen’s expanded behavioral model is to improve the original model’s ability to explain concepts of race and ethnicity and their relation to LTSS use. Inefficiencies identified in the original model include limitations of the “beliefs” construct in capturing the psychosocial factors inherent in race and ethnicity, along with the possibility that the role of race and ethnicity in service use is oversimplified. Using a grounded theory approach, Bradley and colleagues identified how psychosocial factors could be comprehensively described in the context of race/ethnicity and service use in long-term care. Two additional factors that emerged in the expanded model similar to the original model were: enabling factors, and need factors. Domains within these factors described the attitudes, knowledge, and social norms of older adults, along with their perceived control, availability of support, state of financial resources, and objective and perceived need of LTSS. Several themes and their dimensions within each domain encompass the complex interrelationships derived from the three factors. These factors and domains were identified as determinants of service use in the context of intended LTSS use. In building the expanded model, Bradley and colleagues elicited perceptions of LTSS intent to use nursing home, assisted living, adult daycare, home care, and informal care services among African-American and White participants who may have used LTSS or had involvement with the care of family or friends who had used such services [14]. The factors and their domains from this expanded framework are defined in more detail below.

#### Psychosocial factors

Previously termed predisposing characteristics in the original Andersen model, psychosocial factors influence decision making of planned or intended behavior and are derived from the Theory of Planned Behavior. These characteristics include four domains: attitudes, knowledge, social norms, and perceived control [14].

#### Enabling factors

Enabling factors relate to having appropriate community and individual-level resources necessary for accessing care. The availability of support and facilities where people live and work along with the ability to access this support (e.g., funds) are critical. Care that is needed and sought might be restricted because of enabling factors (such as availability or supply of services), ability to pay, or discrimination. These factors can impact the utilization of healthcare services overall [14].

#### Need

Need relates to how individuals view their own health and functional state or how someone else describes their health and functional needs (e.g., healthcare provider). One’s perception of need can be influenced positively or negatively by his or her perceived severity of health, access to health education programs, and availability of financial resources and/or incentives [14].

### Study design

We performed a secondary data analysis of existing baseline qualitative data. The parent study, from which this current research was derived, consisted of a longitudinal cohort design aimed to explore health-related quality of life for older adults utilizing LTSS. At the start of services, a convenience sample of 470 older adults was enrolled between 2006 and 2010 from nursing homes (NHs; *n* = 158), assisted living (ALs; *n* = 156), and home and community-based services (HCBS; n = 156) located in the Philadelphia, New Jersey, and New York metropolitan areas. Participants had to meet the following study inclusion criteria: enrollment within 60 days of the start of LTSS, ≥ 60 years of age, not prior recipients of LTSS; and could communicate in English or Spanish. Older adults with severe cognitive impairment (assessed using the Mini Mental State Examination, [MMSE]; score < 12) [16] were excluded from the parent study. The MMSE ranged from: 0–30; normal to little impairment: 24–30, mild impairment: 20–23, moderate impairment: 12–19) [16]. Representatives from the LTSS site assisted with recruitment of older adults who potentially met study eligibility criteria. Brochures with study and eligibility information were also distributed to this group within the sites. The methods used in the parent study are described in greater detail elsewhere [17]. In regards to our secondary analysis of these data, we employed no exclusion criteria.

### Data collection

One-on-one in-person structured interview sessions took place with the administration of a survey at the respective facility or in a participant’s home in a private room and lasted approximately 1–2 h. Interviews took place at baseline and quarterly for two years. Interviewers were trained to ensure consistent data collection procedures (week-long session with a co-Investigator with survey and qualitative interviewing expertise). During this training, interviewers were required to observe the survey being administered, practice with a test volunteer participant, and be observed administering the survey to actual participants. Written informed consent or assent was obtained from each participant prior to the start of the study. For LTSS recipients who wanted to enroll but who scored < 23 on the MMSE, written assent was obtained from the participant and their designated responsible party provided written consent. Additional details regarding this interview have been published elsewhere [17]. The Institutional Review Boards at University of Pennsylvania, Philadelphia Veterans Administration Medical Center, and the Visiting Nurse Services of New York approved the study’s methods.

### Demographic characteristics

Demographic characteristics were extracted from the survey and included race (i.e., African-American, White, Other [those who were Hispanic and did not select a race, Hawaiian, Asian, American Indian/Alaskan Native, or missing]), ethnicity (Hispanic, non-Hispanic), age, education (how many years of school the participant completed or highest degree attained converted to years), gender (male, female), marital status (single-never married, married, widowed, divorced, separated), religiousness/spirituality (how religious/spiritual the participant said they were rated on a 4-point scale: 1-not at all religious/spiritual, 2-not too religious/spiritual, 3-fairly religious/spiritual, 4-very religious/spiritual), number of chronic conditions (a total count of chronic conditions identified via chart review using ICD-9/ICD-10 codes e.g., diabetes, heart failure, hypertension), MMSE (measure of cognition assessed via a series of yes or no questions [0 = incorrect, 1 = correct], lower scores indicated poorer cognition and ranged from: 12–30; normal to little impairment: 24–30, mild impairment: 20–23, moderate impairment: 12–19) [16]), number of children living, and LTSS type (NH, AL, HCBS).

### Outcome of interest- reason for LTSS use

At the end of the multi-item survey, two open-ended questions collected at baseline were analyzed for our secondary data analysis: “Can you tell me why you moved to [INSERT NAME OF AL/NH]?” for those in an AL or NH setting or “Can you tell me why you are receiving services from [INSERT NAME OF PROGRAM]?” for those in a HCBS setting. Probe questions for those in AL or NH included: “Was there a reason why you moved to [INSERT NAME OF AL/NH]?” and “Did something change that resulted in your need to move to [INSERT NAME OF AL/NH]?” For those in HCBS, probes included: “Was there a reason why now you are receiving these services?” and “Did something change that resulted in your need for these services?” Interviews were conducted in English or Spanish. Interviewers were asked to write verbatim what was said by the participant at the time of the interview. To confirm the trustworthiness of the data, interviewers were trained to repeat what they heard back to the participant and ask if it was correct. Data were entered into a database with only the assigned identification number and stored on a password-protected server. Scanned copies of the initial interview were reviewed for accuracy of the database.

### Data analysis

We used three approaches to analyze the open-ended outcome of interest data [18]. The first was a conventional content analysis approach to allow codes to flow freely from the data. The second was reviewing with a second researcher (KBH) the codes derived from our conventional content analysis and then mapping them to original dimensions within Andersen’s expanded model using a directed analysis approach. Dimensions were codes that fell under major themes. For codes that failed to map appropriately to the expanded model, new model dimensions were created (newly emergent). The same two researchers worked together to discuss newly emerging dimensions and finalize codes. Once initial codes were finalized, one researcher (JLT) primarily coded the data according to the dimensions and themes that had been created. More than 10% of the data were double coded by JLT and KBH and discrepancies were resolved during routine meetings until a Kappa agreement of 90% or greater was achieved [19]. To add validity to our findings, we instituted a classical content analysis approach that consisted of quantifying codes (counting the number of times each code is utilized) [20]. This technique is useful when there are a lot of codes and one wants to decipher which codes are used the most leading to which ones might be the most important. This approach also allowed us to decipher the frequency and proportion of who was saying what. NVivo 11 qualitative software was used to facilitate the coding and organization of the data.

## Results

Among the 470 older adults who participated in the parent study, 464 (99%) provided responses to the open-ended survey questions. A similar number of older adults were represented in each LTSS setting and, on average, were 81 years old. A total of 51% of the study participants were White, 34% African-American, and 14% Other. Ethnically, 20% of the older adults identified as being Hispanic. The majority of Hispanics (90%) resided in HCBS while the majority of those residing in AL were White (94%). Those in NHs were nearly two-thirds African-American and one-third White. Further descriptive characteristics of the study sample can be found in Tables 1 and 2.

**Table 1:** Participant sociodemographic characteristics by race

		NH (*n* = 154)			AL (*n* = 156)			HCBS (*n* = 151)		
	Total (*n* = 464)	White (*n* = 50)	African-American (*n* = 96)	Other (*n* = 8)	White (*n* = 146)	African-American (*n* = 3)	Other (*n* = 7)	White (*n* = 40)	African-American (*n* = 60)	Other (*n* = 51)
Individual Characteristics										
Age, *years*	81.0 ± 8.68	79.1 ± 9.61	76.3 ± 8.30	68.9 ± 7.72	86.5 ± 6.03	82.0 ± 7.00	90.0 ± 2.45	81.8 ± 6.13	78.9 ± 8.04	78.5 ± 8.19
Education, *years*	11.9 ± 4.43	13.3 ± 3.25	11.2 ± 2.53	11.6 ± 1.30	14.9 ± 3.39	15.7 ± 0.58	15.6 ± 2.94	9.9 ± 5.48	9.5 ± 3.46	7.1 ± 4.54
Gender (Female)	330 (71.1%)	32	54	5	125	2	4	27	41	40
Marital status										
Single	49 (10.6%)	11	10	4	11	0	0	2	3	6
Married	93 (20.1%)	10	16	0	30	0	2	8	10	16
Widowed	25 (5.4%)	3	5	0	1	0	0	0	12	4
Divorced	56 (12.1%)	5	21	1	8	1	0	9	7	4
Separated	240 (51.8%)	21	44	3	96	2	5	21	28	20
Religiousness/spirituality	3.0 ± 0.84	2.8 ± 0.89	3.2 ± 0.75	3.6 ± 0.74	2.8 ± 0.79	3.3 ± 0.58	2.4 ± 0.79	3.0 ± 0.97	3.15 (0.83)	2.9 (0.78)
No. of chronic conditions	8.6 ± 3.9	11.5 ± 4.89	9.0 ± 3.03	9.1 ± 2.17	9.4 ± 3.92	7 ± 3	10.4 ± 4.58	7.6 ± 3.53	6.8 (3.18)	5.4 (2.25)
MMSE	24.0 ± 4.3	24.1 ± 4.33	21.1 ± 4.86	20.9 ± 4.85	25.5 ± 3.83	24.7 ± 1.53	23.6 ± 2.76	24.9 ± 2.83	23.9 (3.4)	24.8 (3.74)
No. of children living	2.8 ± 2.6	2.1 ± 1.92	3.1 ± 3.55	2.63 ± 1.92	2.4 ± 1.67	2 ± 1.00	2.1 ± 1.46	3.3 ± 2.45	3.3 (2.79)	3.7 (2.81)

*NH* nursing home, *AL* assisted living facility, *HCBS* home and community-based service, *MMSE* mini mental state examination; Hispanic ethnicity and race were not mutually exclusive in this table; other = those who were Hispanic and did not select a race accounted for 63% of other, the remaining were Hawaiian, Asian, American Indian/Alaskan Native, or missing; 3 Hispanic participants had missing data for race- participants did not provide any information on any race; 2 in the NH and 1 in the HCBS. One participant had missing data for marital status, education, MMSE, and no. of children. Values are presented as mean ± standard deviation, n(%) or n

**Table 2:** Participant Sociodemographic Characteristics by Ethnicity

	NH (*n* = 156)		AL (*n* = 156)		HCBS (*n* = 152)	
	Hisp (*n* = 6)	Non-Hisp (*n* = 150)	Hisp (*n* = 3)	Non-Hisp (*n* = 153)	Hisp (*n* = 82)	Non-Hisp (*n* = 70)
Individual Characteristics						
Age, *years*	80.2 ± 9.56	76.7 ± 8.97	85.3 ± 1.53	86.6 ± 6.04	78.3 ± 7.04	80.9 ± 8.19
Education, *years*	10.2 ± 2.40	11.9 ± 2.92	17.3 ± 3.51	14.9 ± 3.3	6.8 ± 4.49	11.3 ± 3.40
Gender (Female)	3	88	3	128	61	47
Marital status						
Single*	0	26	0	11	9	3
Married	1	26	0	32	26	8
Widowed	0	8	0	1	4	12
Divorced	2	25	0	9	12	8
Separated	3	65	3	100	30	39
Religiousness/spirituality	3.3 ± 0.52	3.1 ± 0.84	2.0 ± 1.00	2.8 ± 0.79	3.0 ± 0.82	3.07 ± 0.90
No. of chronic conditions	8.5 ± 2.43	9.9 ± 3.88	12 ± 2.00	9.4 ± 3.96	5.8 ± 2.56	7.4 ± 3.49
MMSE	17.3 ± 4.37	22.2 ± 4.81	27 ± 3.00	25.4 ± 3.79	24.8 ± 3.19	24.1 ± 3.61
No. of children living	1.8 ± 1.33	2.7 ± 3.11	2 ± 1.73	2.3 ± 1.65	3.9 ± 2.87	2.8 ± 2.40

*NH* nursing home, *AL* assisted living facility, *HCBS* home and community-based service, *MMSE* mini mental state examination, MMSE score ≥ 23 indicated no cognitive impairment to very mild cognitive impairment; score of 12–22 indicated mild to moderate cognitive impairment. Values are presented as mean ± standard deviation or n

The three Andersen behavioral health service use factors—psychosocial, enabling, and need—remained factors in our analyses reflecting aspects of the older adults’ reasons for LTSS use. However, a few dimensions within the factors were more salient than others. We identified four new dimensions through conventional content analysis: losses and changes [psychosocial] and tangible support, capability to provide informal support, and accessibility of informal support [enabling]). Sixteen dimensions that were originally in the expanded model did not emerge during our directed analysis (dimensions that reflected <=1 respondent, e.g., interpersonal skill, home ownership [see Table 3 for entire list]). Nurses and other professionals were added as additional referents, caregiver expectations were modified to expectations of care, decision makers were split up into many layers based on who was making the decision or how the decision was being made and physical and cognitive need were merged to functional health.

**Table 3:** Newly emergent and modified dimensions to Andersen’s expanded behavioral model (insert after third paragraph in results)

Factors	Domains	Major Themes and their Dimensions
Psychosocial	Attitudes	Care Providers
		Technical Expertise
		Affordability
		Perceived expense
		Social Environment
		Social interaction
		Activity Level
		Familiarity
		Diversity
		Self-determination
		Independence
	Knowledge	Content and Amount of Information
		Sources of Information
		Family/friends/^self-
		Medical professionals
		Accessibility of Information
	Social Norms	Referents
		Spouse, ^family, children, ^self
		Friends, neighbors
		Social workers, ^nurse, clergy, doctors, lawyers, ^other professionals, ^they
		Relevant Norms
		Family burden
		*Expectations of care
		^Losses and changes
	Perceived Control-	Role of Choice
		Decision maker-split into 4 layers
		^Autonomous decision
		^Collaborative decision
		^Paternalistic decision
		^Placement
		^Unsure or forgot how decision was made
		Alternatives
		Planning for Future Needs Financial planning
		Psychological planning
Enabling	Availability of Support	Formal Services
		Openings at facilities/waiting lists
		Proximity
		^Tangible Support
		Informal Support
		Ability to provide support- split into 2 layers
		Proximity
		Capability
		^Accessibility of informal support
	Financial Resources	Financial Well-being
		Assets
		Protection against Risk
		Insurance
Need	Objective/Perceived	Degree of Disability
		*Functional health
		Duration of Disability
		*Functional health

*Note*: Dimensions with ^ were newly emerging; dimensions with * were newly modified (<=1 respondent)

We created a conceptual framework depicting newly emergent (not present in expanded model) and modified dimensions (revised from expanded model) to Andersen’s expanded behavioral model after performing our conventional analysis (Table 3). Emerging data were not mutually exclusive to one dimension and could have been coded under multiple dimensions. Classical content analysis revealed the proportions of older adults within each LTSS and from each racial/ethnic background who identified with a specific dimension (Table 4). The classical content analysis results for Hispanic older adults vs. non-Hispanic older adults can be found in Supplemental Table 1. In the following sections, we discuss our findings within the context of LTSS use across racial and ethnic older adult groups.

**Table 4:** Quantification of factors, domain, themes, and dimensions through classical analysis (insert after third paragraph in results)

Major Factors, Domains, Themes and their Dimensions	NH = 154			AL = 156			HCBS = 151		
	White (*n* = 50)	AA (*n* = 96)	Other (*n* = 8)	White (*n* = 146)	AA (*n* = 3)	Other (*n* = 7)	White (*n* = 40)	AA (*n* = 60)	Other (*n* = 51)
PSYCHOSOCIAL	50	99	7	227	5	6	43	63	42
Attitudes	13	17	1	43	0	0	15	14	20
Care Providers	**9**	**11**	**1**	**16**	**0**	**0**	**10**	**6**	**18**
Technical Expertise	9	11	1	16	0	0	10	5	18
Interpersonal Skill	0	0	0	0	0	0	0	1	0
Affordability	**1**	**0**	**0**	**3**	**0**	**0**	**1**	**1**	**0**
Perceived expense	1	0	0	3	0	0	1	1	0
Home Ownership	0	0	0	0	0	0	0	0	0
Social Environment	2	6	0	11	0	0	2	6	1
Social interaction	1	3	0	8	0	0	2	5	0
Activity Level	0	1	0	0	0	0	0	1	1
Familiarity	0	1	0	3	0	0	0	0	0
Diversity	1	1	0	0	0	0	0	0	0
Self-determination	1	0	0	8	0	0	2	1	1
Privacy	0	0	0	0	0	0	0	0	0
Dignity	0	0	0	0	0	0	0	0	0
Independence	1	0	0	8	0	0	2	1	1
Knowledge	0	3	0	4	0	0	7	11	0
Content and Amount of Information	0	0	0	1	0	0	0	1	0
Service types provided	0	0	0	1	0	0	0	0	0
Eligibility rules	0	0	0	0	0	0	0	1	0
Legal/regulatory issues	0	0	0	0	0	0	0	0	0
Financial coverage	0	0	0	0	0	0	0	0	0
Sources of Information	0	3	0	3	0	0	6	9	0
Family/friends/SELF-	0	3	0	3	0	0	1	4	0
Lawyers	0	0	0	0	0	0	0	0	0
Medical professionals	0	0	0	0	0	0	5	5	0
Clergy	0	0	0	0	0	0	0	0	0
Accessibility of Information	0	0	0	0	0	0	1	1	0
Attainability	0	0	0	0	0	0	1	1	0
Comprehensibility	0	0	0	0	0	0	0	0	0
Social Norms	11	20	1	81	2	1	11	20	13
Referents-	4	12	1	42	0	1	10	17	8
Spouse, *FAMILY, children, *SELF	3	7	0	28	0	1	2	6	4
Friends, neighbors	0	1	1	2	0	0	0	0	1
Social workers, *nurse, clergy, doctors, lawyers, *other professionals, *they	1	4	0	12	0	0	8	11	3
Relevant Norms	7	8	0	39	2	0	1	3	5
Family burden	1	2	0	11	0	0	1	0	0
Expectations of care	3	1	0	9	1	0	0	2	5
*Losses and changes	3	5	0	19	1	0	0	1	0
Perceived Control	26	59	5	104	3	5	10	18	9
Role of Choice	25	59	5	89	2	4	5	11	7
Decision maker-split into 4 layers	21	51	4	79	2	4	3	9	2
Autonomous decision	3	1	1	32	1	0	1	4	0
Collaborative decision	3	4	0	23	0	1	0	1	0
Paternalistic decision	9	19	0	18	0	2	2	2	1
Unsure/forgot how decision was made	3	3	2	3	1	1	0	2	0
Placement	3	24	1	3	0	0	0	0	1
Alternatives	4	8	1	10	0	0	2	2	5
Planning for Future Needs	1	0	0	15	1	1	5	7	2
Financial planning	0	0	0	0	0	0	0	0	0
Psychological planning	0	0	0	0	0	0	0	0	0
ENABLING	10	19	1	37	2	1	9	12	9
Availability of Support	8	16	1	37	3	1	8	10	9
Formal Services	4	8	0	23	1	1	7	9	6
Openings at facilities/waiting lists	2	0	0	4	0	0	0	2	0
Hours of operation	0	0	0	0	0	0	0	0	0
Proximity	2	4	0	10	0	0	0	0	0
Tangible Support	0	4	0	9	1	1	7	7	6
Informal Support	4	8	1	14	2	0	1	1	3
Willingness to provide support	1	0	1	0	0	0	0	0	0
Ability to provide support- split into 2 layers	2	7	0	13	1	0	1	1	2
Proximity	0	0	0	2	1	0	0	0	0
Capability	2	7	0	11	0	0	1	1	2
Accessibility of informal support	1	1	0	1	1	0	0	0	1
Financial Resources	2	3	0	0	0	0	1	1	0
Financial Well-being	0	2	0	0	0	0	1	0	0
Income	0	1	0	0	0	0	0	0	0
Assets	0	1	0	0	0	0	1	0	0
Protection against Risk	2	1	0	0	0	0	0	1	0
Insurance	2	1	0	0	0	0	0	1	0
NEED	18	47	3	70	1	2	15	30	32
Objective/Perceived Need	18	47	3	70	1	2	15	30	32
Degree of Disability									
Functional health	18	44	3	70	1	2	15	30	32
Duration of Disability									
Functional health	0	3	0	0	0	0	0	0	0

*Note*. Three Hispanic participants had missing data for race- participants did not provide any information on any race; Placement included transferred/sent from facility/taken from home/put. Hispanic ethnicity and race were not mutually exclusive in this Table. *NH* nursing home, *AL* assisted living, *HCBS* home and community-based service; *AA* African-American. *Newly emergent dimensions

### Psychosocial factors for determinants of long-term services and support use

Attitudes (*n* = 123), knowledge (*n* = 25), social norms (*n* = 160), and perceived control (*n* = 239) are all constructed under the psychosocial determinant factor in Andersen’s expanded behavioral model. Each of these domains was cited by 5% or more of participants.

#### Domain of attitudes

Attitudes consist of participants’ views on LTSS use related to the themes of: care providers, affordability, social environment, and self-determination.

Care providers were those who delivered formal or informal care. They were desired for their ability to provide technical expertise (e.g., based on prior training and experience or knowledge on how to handle emergencies) or interpersonal skill (trustworthiness, compassion, listening, and communication skills) to older adults in need of care. Over 20% of the 464 participants discussed their technical expertise needs that prompted LTSS use. The frequency of responses to this theme varied across racial groups; Other (*n* = 19/66, 29%); White (*n* = 35/236, 15%); and African-American older adults (*n* = 16/159, 10%). Twenty-nine percent of Hispanic older adults reported requiring some form of technical expertise (*n* = 26/91). Common among participants was a need for LTSS as their medical condition worsened. Specifically, one Hispanic participant using HCBS relayed the need for a provider who could “monitor [their] diabetes and hypertension…[and provide] physical therapy.”

Being able to afford services was also important to one’s actual use of LTSS. These views were guided by the perceived expense of services and the impact LTSS use had on home ownership. Concern about affordability was discussed primarily among those who were White and receiving care in AL (*n* = 3). For example, one White participant noted the desire and opportunity to save money as having the most influence on her decision: “AL was cheaper than independent [living] due to not needing a car.” Another White AL participant described the process of obtaining AL as “I sold my house and wanted to be in a medically safe, spiritual, and semi-affordable environment where people are comparable to me.”

In addition to affordability, the presence of a social environment was central to one’s longing for connections achieved by social interaction, participation in activities, familiarity in surroundings, and diversity of the environment. Among those who commented on social environment, social interaction was most commonly discussed and, particularly among White (*n* = 11/236, 5%) and African-American (*n* = 8/159, 5%) participants who were primarily receiving services via AL and HCBS, respectively. For example, having the opportunity to share spaces with other individuals and be active was necessary, as noted by an African-American participant receiving HCBS: “I love being around people, and to get up and know I’m going somewhere. I was living by myself and went to a community center which I loved. Then I moved and started to come here.”

Social interaction was additionally important when coping with the loss of loved ones, as a White participant receiving care in AL reported: “My husband passed away when we were in independent living. It was too hard to be there without him because I kept thinking of him…. I moved here to meet new people…. I just thought I’d be happier and I am.”

On the other hand, participants discussed their views on self-determination while using LTSS as a need to maintain independence. The majority of these views came from White older adults (*n* = 11/236, 5%) followed by the Hispanic ethnic population (*n* = 3/91, 3%) who primarily received services via AL and HCBS, respectively. For some participants, the choice of LTSS was based on their inability to live independently any longer, while for others, it was to maintain or regain their independence. A White participant in AL described the need for independence as “I was lonely living by myself; afraid of falling or experiencing pain. I wanted to make my own end of life decisions. I wanted to plan the rest of my future and make my own choices…. I like to live elegantly and want my independence.”

#### Domain of knowledge

Acquiring knowledge of the LTSS site was critical for making decisions about LTSS use and being aware of available options. While the content and amount of information did not emerge as a theme, what did emerge was who was providing information specific to LTSS (sources of information). Family, friends, study participants as well as health professionals were primarily discussed as key sources of information among the African-American older adult subgroup (*n* = 12/159, 8%). Family members typically visited different LTSS options and then shared what they learned with older adults. When describing this process, an African-American HCBS participant particularly noted, “My niece told me about it and I came and tried it and liked it.” Older adults across the White, African-American, and Hispanic racial/ethnic groups similarly reported receiving letters or cards or receiving information from their hospitals. Participants most commonly mentioned social workers and doctors as the health professionals who communicated with them about LTSS use.

#### Domain of social norms

Social norms consist of two themes: referents and relevant norms. Referents were defined in Andersen’s expanded behavioral model as decision makers who were considered legitimate sources of authority; however, in our analyses, this theme closely mirrored the sources of information theme. Therefore, to differentiate between the two, we further defined the referent dimension as a process that appears to be collective; that is, the participant was referred to a particular setting, it was suggested to them, and/or the referent had knowledge or experience with the LTSS site. Referents included friends, family, and members of the healthcare and professional team (i.e., social workers, clergy, doctors, and lawyers). “Nurses” and “Other Professionals” were added as additional referents from our analyses. White older adults more commonly reported spouses, family, and children serving as referents to services, while African-Americans discussed the healthcare and professional team (e.g., lawyers) serving as referents. For example, an African-American participant described a reference to their current NH along the lines of “The social worker said it was better for my family.”

Relevant norms were aspects that had direct bearings on one’s choice to use LTSS. The dimensions within this theme were comprised of the following norms: family burden, expectations of care, and losses and changes; the second norm was modified from care expectations, and the third norm was a newly emerging dimension. Relevant norms were primarily discussed by White participants. Regarding family burden, a concern was not just for burdening one’s family but also oneself and others outside of the family. A White participant using AL discussed her desire to avoid family burden as follows: “I wasn’t able to take care of my daily needs and my fatigue level caused me to sleep all of the time and miss meals and meds. It was taking too much of a toll on my daughter and it wasn’t fair to her.”

Expectations of care focused on participants’ goals while using LTSS. These goals were as generic as “to get better” or more specific as the need for “better communication.” The norm “losses and changes” was added as part of a relevant aging norm that consisted of experiences with death, illness, and decreased ability to care for the home. A White AL participant discussed the experience with losses and changes in functional status requiring subsequent LTSS use: “I became ill and my husband was sick then too. We couldn’t really care for each other then. Upon getting better, our kids felt it was time to move and give up the house that required too much care so we began making arrangements. We weren’t expecting to come to a place like this but needed it.”

#### Domain of perceived control

Perceived control describes the participants’ involvement in LTSS decisions made on their behalf. This domain specifically consists of the two themes: participants’ role of choice in LTSS use and prior planning for future needs and two dimensions: decision maker and alternatives. Because many players affected the older adults’ role of choice of LTSS use, the dimension of “decision makers” was broken into five subdimensions: autonomous, collaborative, paternalistic, placement (transferred/sent from facility/taken from home/put), and unsure or forgot.

One’s role in the decision making of LTSS use differed according to race/ethnicity and LTSS type. A greater proportion of White participants (*n* = 62/236, 26% [White older adults] vs. *n* = 11/159, 7% [African-American older adults]) discussed this decision as being autonomous or collaborative and used AL services, while a greater proportion of African-American participants (*n* = 45/159, 28% [African-American older adults] vs. *n* = 35/236, 15% [White older adults]) discussed this decision as being paternalistic or part of a “placement” process and used NH services.

In making the decision autonomously, a White participant discussed the process as being a combination of factors, but at the end of the day stated feeling this way: “I was having knee replacement surgery and expected that I wouldn’t recover enough to move back to independent living. The move was my choice. I didn’t want to be in independent living. I don’t think I would have moved if it hadn’t been for the surgery. I also knew that I could spend time post-surgery in [a] NH before being able to move back to AL.” Another White participant discussed the decision for LTSS use being collaborative with input from family; as she recalled, “I had a couple of falls in Florida and the kids felt it was time to move. We all agreed that this was a good place for my husband and I. We all agreed he was a little too much for me to handle alone.” Alternatively, an African-American participant in a NH discussed having no role in the decision making of LTSS use and the decision being paternalistic: “They felt that I needed to come here. This was not a decision I made. The doctors talked my family into moving me here.” Participants additionally discussed LTSS use as a process initiated and carried out by the hospital or medical professional coded in this study as “placement.” A NH African-American participant stated, “When I was hospitalized, they thought I should be in rehabilitation. When you can’t walk or do nothing, this is where they put you.”

Alternatives was characterized by changes to care needs, preferences, and options that influenced LTSS use. These alternatives ranged from the older adult having no other place to go, the older adult leaving unsatisfactory conditions and looking for a better way of life, or the older adult no longer being able to care for himself or herself. For example, an African-American participant in the NH described it as follows: “Because I knew I would get better services here than living on my own—doctors, laundry, food, it’s 24/7 here and you don’t have to worry about the aide not making it in.”

Planning for future needs comprised the second theme under perceived control. This theme described long-term decision making as a process unfolding over time and consisted of two dimensions: financial planning and psychological planning. Financial considerations such as affordability of LTSS and one’s state of finances were important to future planning, along with one’s psychological preparation that followed trends or beliefs in LTSS use as a preventive measure or a product of age. For one White participant, this trend in psychological preparation was described as a process dictated by the community: “The community pushed me to move. At my age, it seemed like the time had come to be sensible and accept more help. They had asked me a while ago and I wasn’t ready until now.”

### Enabling factors for long-term services and support use

The enabling factor consisted of acquiring family and community resources and the accessibility of those resources, including availability of support and financial resources.

#### Domain of availability of support

Characteristics of the availability of support, both formal and informal, remained emergent with LTSS use. Support was not always available in the location when the older adult wanted it, however. To be considered for LTSS, some participants had to place their names on waiting lists, as one White AL participant noted: “My name was on the waiting list. They called me and offered me this. That made me start thinking perhaps it was a good time.”

Proximity was described in several forms and could have been related to the proximity of services, meals, activities, or care within or from the home, community, or facility. For example, a White participant in AL stated, “The independent housing I was in was very far away. It was hard to walk to the main building after I had my hip operation.” Proximity also took into account the proximity of family, as another White participant in a nursing home explicitly stated: “I wanted to be near my daughter. Family comes first.”

Tangible support (newly emerged) included the need for everyday physical support such as transportation, meals, and housework. An African-American participant using HCBS described this need for support as follows: “I have pain in my left knee and left side of hip. I’m always in pain and I feel very sad most of the time. It affects my ability to do housework and cook for myself so I’m losing weight. I’m afraid to travel by myself to the doctor. I need home assistance.”

The theme of informal support included willingness to provide support, ability to provide support (further broken down to capability [newly emerged] or proximity of the informal support), and accessibility of informal support (newly emerged). Willingness was described as a family member simply being okay with providing care to the older adult. A NH participant part of the Other racial group discussed her daughter’s unwillingness to provide support by stating, “My daughter wants to take her home back and I understand. I was in a room with my two grandkids and I wasn’t getting much sleep.” Regarding capability, an African-American participant residing in a NH discussed care limitations as follows: “Because my kids were working and couldn’t take care of me. We decided it was time for them to go on.”

For proximity of informal support, it was uncertain whether the family elected to serve as the participant’s caregivers or the participant simply wanted to be near them. Accessibility to informal support additionally influenced one’s decision for LTSS use. A White woman in AL described this lack of access as follows: “If I were to have gotten ill in independent living, I would have had no one but my younger sister to take care of me which wasn’t appropriate.” Or another participant who self-described as “Other-More than One Race” living in HCBS discussed the influence of children on LTSS use, reasoning: “Because I didn’t have children and I didn’t want [any].”

#### Domain of financial resources

Financial resources as they related to one’s financial well-being (i.e., income, assets) and protection against risks (i.e., insurance) were important in one’s ability to access services, but were less salient to actual LTSS use when compared to other dimensions.

Within this dimension, having insurance and considerations about income were most salient to LTSS use. One Hispanic older adult described the process of securing HCBS through insurance: “Because I have Medicare, they offered it to me when I was sick, they asked me if I would like to have a girl to help at home because I was ill because of my leg.” Alternatively, an African-American NH participant’s use of LTSS was influenced by income, as indicated by the statement, “I needed to be somewhere I could get [financial] assistance.”

### Need

The need factor was the most commonly discussed factor attributed to actual LTSS use. Need primarily focused on the older adults’ objective or perceived need related to their functional health and the degree and duration of their disability. It originated from a wide range of ailments and included difficulty with vision, recent surgeries (e.g., heart, hip/knee replacements), strokes, heart disease, respiratory disease, or issues related to mobility deficits.

A Hispanic White/African-American participant described the complexity of functional need requiring LTSS use: “Because I can’t see, I can’t walk. I don’t have good balance. I need help at home and to be able to go out. I can’t check my blood sugar level because I can’t see.”

## Discussion

In this section, we synthesize our findings reflecting actual LTSS use across racial and ethnic groups compared with findings reflecting intended LTSS use, as described in Andersen’s expanded behavioral model. In our analyses, four new dimensions emerged under psychosocial and enabling factors (i.e., losses and changes [psychosocial] and tangible support, capability to provide informal support and accessibility of informal support [the three latter dimensions fell under enabling]). Interpretations of several existing themes were enhanced based on participants’ responses to actual LTSS use (e.g., sources of information vs. referents, role of choice), while others in the current model were not as salient to our findings (e.g., affordability, content and amount of information, accessibility of information, financial well-being). Variations existed among racial and ethnic groups in several themes depicting decision making, family burden, and technical expertise of the care provider. Our findings convey the importance of understanding actual LTSS use as opposed to intended LTSS use alone and reaffirms the need to consider variations in LTSS actual use as well as intention across racial and ethnic groups and LTSS types.

### Emerging and modified themes

The emergence of the losses and changes theme in our exploration of actual LTSS use and not in intent to use, possibly stems from older adults’ failure to recognize this phenomenon as an imminent issue they will potentially face. Experience of losses and changes indisputably uproots older adults’ ways of life, their resources, and the manner in which they carried out their activities previously, thus signifying their need for LTSS. As described in the literature, the most significant losses and changes to older adults are death of a loved one, physical frailty, and relocation [21]. Amid such losses [22] and changes comes the loss of hope, identity, independence, goals, expectations, and mental stability, along with the impact these losses and changes have on the older adults’ well-being [21, 23–25]. While understanding the role that losses and changes play in LTSS use is key to coordinating appropriate care needs for older adults, the next step is reacting to the losses and changes. Specifically, it is essential to identify how best to recreate meaning in the life of older adults and build up their resiliency [21, 26–28]. Activities such as storytelling, goal setting, bolstering programs, fostering hope, and strengthening spiritual connections and relationships are ways to support resiliency [21, 24, 26, 29]. Healthcare professionals, family, and community support are critical to this role and should be leveraged when looking to address losses and changes experienced by older adults [25].

Tangible support, which is a form of social support and another newly emerging theme in this study, has been defined as the physical provision of needed goods and services to recipients [30]. In our findings, the tangible support that was commonly gained and appreciated as a result of LTSS use included transportation, cooking, housework, and shopping. It was surprising that this form of social support along with others (i.e., emotional support) did not emerge in the original expansion of this model. Because, the provision of social support is associated with health promoting behaviors however, pre- discussions around the availability of such support are warranted.

Regarding other forms of support (i.e., informal support), older adults newly recognized that the availability of informal support was only one component driving LTSS use, but whether that informal support was actually capable of providing needed care for older adults or truly accessible to older adults was another driver of actual LTSS use. This finding further highlights the direction that the provision of informal support is headed and the need for future LTSS planning that includes family and friends.

In addition to including the described newly emerging dimensions, we recommend clarifying between the sources of information (knowledge) theme and the referents (social norms) theme. A person who shares information can also be a person who provides a referent/recommendation at the same time; this duality made it difficult to differentiate between these themes. Broadly recognizing that other professionals (and nurses specifically) are in positions to provide recommendations about LTSS is also necessary to encompass those who are key in connecting LTSS to older adults. The caregiving expectations dimension was modified to expectations of care to capture what one wanted from care now that they are using that LTSS.

### Racial and ethnic differences

The decision-making role emerged as a key process important to older adults when considering LTSS use and it also differed across racial and ethnic groups. To highlight these differences, we broke decision making into additional layers reflecting autonomous decision, collaboration, paternalism, and placement. In Johnson et al.’s (2010) mixed-methods study on participation in NH placement decision making among African-American and White older adults, two themes emerged from their focus groups: “They made the decision” and “We made the decision.” Similar to our findings, the African-American older adults in Johnson’s study (*n* = 7/7) discussed having no or minimal participation in the decision to be placed in a NH (paternalism or placement), while the majority of White participants (n = 7/9) reported total or some participation (autonomous or collaboration). White older adults in Johnson’s study specifically discussed sitting down with family to make the decision or already having made up their mind to be placed in a NH once their health warranted it. African-American participants alternatively discussed being lied to or tricked into NH placement or having social workers and nurses highly involved in the decision to be placed in a NH. These are important findings and similarities across-studies when considering the level of mistrust that might arise among racial/ethnic minorities who are known to have been subjected to multiple forms of disparities by the healthcare system [31, 32]. To this point, racial/ethnic minorities have an extensive history of being manipulated to engage in unethical activities that have subsequently stressed their ability to trust and comply with the healthcare system [31, 33]. Such continued encounters will only cause additional strain further emphasizing the need to include both racial and ethnic minority older adults and their families in all healthcare decisions whenever possible [34] to overcome recollections of domination and coercion.

It is not surprising that the desire to relieve the burden of caretaking from family members was more salient to LTSS use among Whites, when compared to African-Americans. Historically, the use of informal support (i.e., family caregivers) has been a common theme among the racial/ethnic minority population, and even assumed. However, as the availability of informal caregiver support continues to decline for several reasons—the caregivers’ need to work outside the home, the decreased number of children to provide care, and the increasingly complex care needs of sick older adults who require higher levels of supports, conversations around the potential need for LTSS use must be conducted between family members and older adults early so that older adults are adequately prepared for who will be providing the LTSS. Moreover, clearly defining what assistance is available for families to support older adults is also critical. A continued environment where assumptions are held that the African-American and Hispanic community will provide informal support to the older adult population will only perpetuate caregiving burden and fail to provide this community with adequate resources and supports to equitably prepare for more sustainable options.

### Differences across LTSS

The theme around care providers was most salient among those using HCBS—primarily Hispanic older adults. The role of referents and relevant norms (i.e., burden, expectations of care, and losses and changes) in LTSS use was most relevant to AL and HCBS. The participants most commonly discussed the role of control in LTSS use in NHs and ALs, where paternalistic decision making was most common in the former and autonomous decision making was most salient in the latter. It is evident in the literature that maintaining function is a common goal for older adults whereby unsolicited placement in a facility may not be wanted nor in the older adult’s best interest [35]. Our findings highlight the opportunity to evaluate how older adults are involved in the planning of LTSS when it comes to LTSS placement, particularly NHs.

### Themes that did not emerge

Themes that did not emerge as prominently in the present study included affordability, staffing, and self-determination within the attitudes domain, as well as financial resources within the enabling domain.

### Policy implications

Policies directed at LTSS delivery for the older adult population are necessary and must be racially and ethnically inclusive to reflect the experiences of all racial and ethnic groups when accessing and using LTSS. In partnership with various stakeholders, the Centers for Medicare and Medicaid Services (CMS) aim to foster a person-driven LTSS system where those who are experiencing debilitating conditions are able to maintain control of their choice and access to quality LTSS [36]. To meet these aims, it is important to ensure that racial and ethnic minority older adults are also maintaining control of choice. This reality was not evident in this study as well as others, particularly among African-American older adults using NH services [35]. The Older Americans Act has supported Area Agencies on Aging in providing personalized information on the services and supports available in each community and assisting in decision making through Eldercare Locator. To further support knowledge and decision making, the Department of Health and Human Services and the Veteran Affairs have developed a one-stop shop for LTSS called “no wrong door” systems. Older adults selecting a nursing facility or home healthcare agency can also obtain information on the quality of CMS services through their Nursing Home Compare or Home Health Compare websites, respectively.

While these described initiatives aim to support LTSS decision making, it has been important to scrutinize how interpretable the information provided is and how sensitive are those supporting the decision-making process to the needs and wants of older adults, specifically those from racial and ethnic minority backgrounds [37]. For example, bias may be implicit when healthcare professionals (e.g., social workers, case managers, and providers) arrange LTSS for racial and ethnic minority older adults and fail to regard LTSS planning as a collaborative approach, including input from the older adults. Instead, their approach resorts to a paternalistic process [38].

Healthcare professionals’ paternalistic role in LTSS decision making could also be the result of restrictions imposed by insurance coverage. Medicaid, which is the primary payor for LTSS and whose beneficiaries are largely African-American and Hispanic older adults, has several restrictions to site of care which can further limit the role of choice among racial and ethnic minority older adults. Families are additionally seen assuming a paternalistic role in LTSS planning [35]. While support from both healthcare professionals and families is essential, racial and ethnic minority NH residents are routinely sent to NHs of lower quality and poor performance. Therefore, when considering NH placement for racial/ethnic minority residents, it is imperative that these healthcare professionals and family members be equipped with the tools, education, resources, and sensitivity to make the best placement decisions for racial and ethnic minority residents while involving them and considering their finances and health needs. Community health workers and *Promotores* have been essential in improving healthcare access and outcomes among the African-American and Hispanic community because these community workers understand the needs of African-Americans and Hispanics and facilitate with navigating the healthcare system [39–41]. Models such as these benefit racial and ethnic minority groups, assist with barriers to access and utilization of quality healthcare services, and reduce cost expenditures [5].

Lastly, differences in access to AL and NH by race/ethnicity must be addressed. Between the years 1999 and 2008, there was a surge in the number of minority older adults living in NHs (54.9% increase in Hispanic older adults and 10.8% increase in African-American older adults) while a decline of White older adults in this setting was evident (10.2%) [8]. The researchers conclude that the increased use of NHs by African-American and Hispanic older adults may be the result of unequal access to more desirable options for LTSS such as HCBS and AL [8]. To speak to this point, a 2019 study examining 442,018 African-American and White Medicare beneficiaries residing in AL across the US found White older adults to make up 95% of the sample while African-American older adults only made up 5% [9]. The disparate access to AL may be shaped by one’s ability to afford these services which typically require private payment/insurance [42]. Racial/ethnic minority older adults more commonly utilize Medicaid payment for LTSS and often live in lower socioeconomic areas where availability of AL is limited in these areas [43–45]. When African-American and Hispanic older adults are noted to reside in AL, they are found to be in more lower quality AL and live in AL with fewer White older adults and more dual eligibles [9, 42]. Emphasis on creating more equitable pathways to desirable quality care is necessary.

### Strengths and limitations

This study has several strengths and limitations. The first strength lies in the methodology that was used to analyze the qualitative data. Our multi-analytical approach allowed us to map similar dimensions and themes to Andersen’s expanded behavioral model (directed analysis), analyze newly emerging themes (conventional analysis), and understand not only the frequency of codes but who was saying what and from which LTSS type (classical analysis). The second strength emerged from our data. We had a comprehensive data set of 464 older adults who were newly enrolled to LTSS, thereby increasing their likelihood of recalling why they were using LTSS. The third strength was the diversity of our sample, which was equally dispersed over the three LTSS types, came from three different states, had nearly 50% who identified as either African-American and/or Hispanic, and consisted of older adults ranging from no cognitive impairment to moderate cognitive impairment (those with cognitive impairment are typically excluded from research).

A limitation is the need for caution when interpreting some of our racial and ethnic differences results. LTSS use can also be based on one’s socioeconomic status and not so much on race and ethnicity, as was probable in this study. For example, the majority of those in AL were White older adults who were also self-pay, while the majority of African-American older adults were in NHs and mostly Medicaid (67%, data not shown). Their source of payment could have played a role in their self-identified ability to be part of decision making in actual LTSS use as described earlier. Nonetheless, disparities in LTSS options have been proposed and warrants further investigation into how best to create equitable access to desired LTSS care despite one’s economic status and race and ethnicity [8, 9, 13]. Lastly, because this is a secondary data analysis of previously collected data, our analysis was only limited to two questions related to reasons for actual use of LTSS. It is possible that there could have been additional questions posed to understand LTSS use as it related to the Andersen Expanded Model.

## Conclusion

Through this in-depth analysis of qualitative data with older adults who provided context to why they were using LTSS, several factors associated with *actual* use of LTSS were identified. Using multiple qualitative approaches, we found consistent evidence for many important factors that may help explain some reasons for *actual* LTSS use among older adults and specific to race and ethnicity and LTSS type. Moreover, this work validates Andersen’s earlier work and provides insights to the experience of older adults in different LTSS settings. These findings led to future implications for policy and research. Finally, we discussed how Andersen’s expanded model may be adapted to represent constructs that reflect *actual* use of LTSS when considering a diverse group of older adults.

## Data Availability

The datasets used and/or analyzed during the current study are available from the senior author, Mary Naylor, at naylor@nursing.upenn.edu upon reasonable request.

## Abbreviations

ADL: Activities of Daily Living
AL: Assisted Living
HCBS: Home & Community-Based Service
IADL: Instrumental Activities of Daily Living
LTSS: Long-term Services and Supports
MMSE: Mini-Mental State Examination
NH: Nursing Home
